# 1429. Real-World Study of Patients with Uncomplicated Urinary Tract Infection in the United States: High-Risk Comorbid Conditions and Burden of Illness

**DOI:** 10.1093/ofid/ofab466.1621

**Published:** 2021-12-04

**Authors:** Madison T Preib, Alen Marijam, Fanny S Mitrani-Gold, Daniel C Gibbons, Xiaoxi Sun, Christopher Adams, Ashish V Joshi

**Affiliations:** 1 STATinMED Research, Ann Arbor, MI, USA, Ann Arbor, Michigan; 2 GlaxoSmithKline plc., Collegeville, PA, USA, Collegeville, Pennsylvania; 3 GlaxoSmithKline plc, Collegeville, PA, USA, Chicago, Illinois; 4 GlaxoSmithKline plc., Brentford, Middlesex, UK, Brentford, England, United Kingdom

## Abstract

**Background:**

Urinary tract infections (UTIs) are associated with significant morbidity and economic burden, particularly in the elderly and patients with comorbidities. We used real-world data (RWD) to assess healthcare resource use (HRU) and costs in patients with uncomplicated UTI (uUTI) and high-risk comorbid conditions in the US.

**Methods:**

This was a retrospective cohort study (IBM MarketScan RWD, commercial/Medicare Supplemental claims January 1, 2014–December 31, 2017) of females ≥ 12 years of age with uUTI who had an oral antibiotic prescription ± 5 days of uUTI diagnosis (index date) and continuous health-plan enrollment for ≥ 1 year pre-/post index date. Five high-risk cohorts and matched-control cohorts (baseline age, region) were identified: controlled type 2 diabetes (T2D), mild/moderate chronic kidney disease (CKD), recurrent UTI (rUTI), elderly (ELD), and postmenopausal (PMP) (**Table 1**). Sample sizes were balanced via random match selection (1:5 case:control). uUTI-related HRU and costs were compared between cases and controls (index episode/1-year follow-up) using multivariable generalized linear models.

Table 1. Cohort assignment for high-risk cohorts and controls

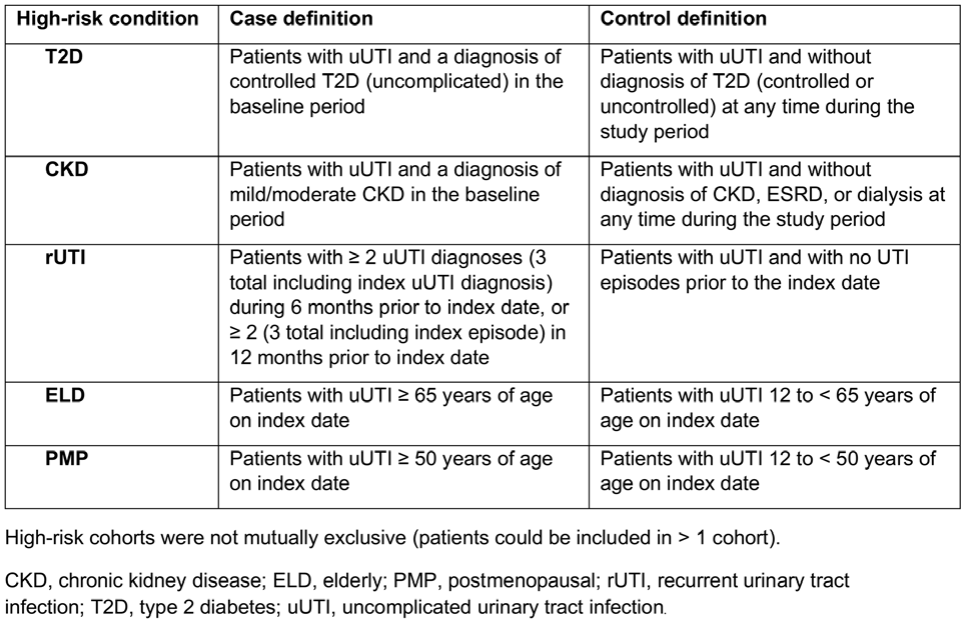

**Results:**

Of 339,100 patients with uUTI, case/control cohorts comprised T2D, n=15,423/n=77,115; CKD, n=1041/n=5205; rUTI, n=7937/n=39,685; ELD, n=23,666/n=118,330; and PMP, n=105,608/n=211,216 patients. HRU trends across cohorts varied. During 1-year followup, outpatient visits were significantly different for cases versus controls in the T2D, rUTI, and PMP cohorts (p ≤ 0.0079), with higher case than control values in the rUTI and PMP cohorts; pharmacy claims were significantly higher for rUTI, ELD, and PMP cases, and inpatient visits were significantly higher for ELD and PMP cases, versus controls (all p < 0.0001; Table 2). Adjusted total uUTI-related costs (emergency room + outpatient + pharmacy) were significantly different (p < 0.0001) for cases versus controls at index episode and during follow-up in all cohorts except CKD: case values were higher than controls at index episode and during follow-up in the T2D cohort, and during follow-up in the rUTI and ELD cohorts (Table 3).

Table 2. uUTI-related HRU* for cases versus controls according to high-risk cohort

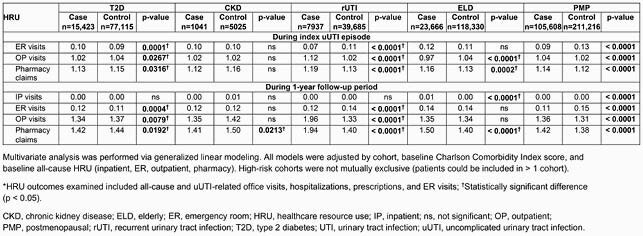

Table 3. uUTI-related costs* for cases versus controls according to high-risk cohort

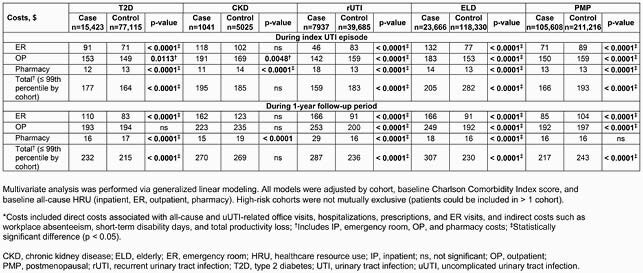

**Conclusion:**

Females in some high-risk case cohorts had higher uUTI-related HRU and costs versus controls. Further studies of relationships between comorbidities and uUTI burden are needed.

**Disclosures:**

**Madison T. Preib, MPH**, **STATinMED Research** (Employee, Former employee of STATinMED Research, which received funding from GlaxoSmithKline plc. to conduct this study) **Alen Marijam, MSc**, **GlaxoSmithKline plc.** (Employee, Shareholder) **Fanny S. Mitrani-Gold, MPH**, **GlaxoSmithKline plc.** (Employee, Shareholder) **Daniel C. Gibbons, PhD**, **GlaxoSmithKline plc.** (Employee, Shareholder) **Xiaoxi Sun, MA**, **STATinMED Research** (Employee, Employee of STATinMED Research, which received funding from GlaxoSmithKline plc. to conduct this study) **Christopher Adams, MPH**, **STATinMED Research** (Employee, Employee of STATinMED Research, which received funding from GlaxoSmithKline plc. to conduct this study) **Ashish V. Joshi, PhD**, **GlaxoSmithKline plc.** (Employee, Shareholder)

